# A machine learning and deep learning-based integrated multi-omics technique for leukemia prediction

**DOI:** 10.1016/j.heliyon.2024.e25369

**Published:** 2024-02-01

**Authors:** Erum Yousef Abbasi, Zhongliang Deng, Qasim Ali, Adil Khan, Asadullah Shaikh, Mana Saleh Al Reshan, Adel Sulaiman, Hani Alshahrani

**Affiliations:** a State Key Laboratory of Wireless Network Positioning and Communication Engineering Integration Research, School of Electronics Engineering, Beijing University of Posts and Telecommunications, Beijing, China; b Department of Software Engineering, Mehran University of Engineering and Technology, Jamshoro, Pakistan; c Department of Information Systems, College of Computer Science and Information Systems, Najran University, Najran, 61441, Saudi Arabia; d Scientific and Engineering Research Centre, Najran University, Najran, 61441, Saudi Arabia; e Department of Computer Science, College of Computer Science and Information Systems, Najran University, Najran, 61441, Saudi Arabia

**Keywords:** Multi-omics, Genomics, Machine learning, Deep learning, Leukemia

## Abstract

In recent years, scientific data on cancer has expanded, providing potential for a better understanding of malignancies and improved tailored care. Advances in Artificial Intelligence (AI) processing power and algorithmic development position Machine Learning (ML) and Deep Learning (DL) as crucial players in predicting Leukemia, a blood cancer, using integrated multi-omics technology. However, realizing these goals demands novel approaches to harness this data deluge. This study introduces a novel Leukemia diagnosis approach, analyzing multi-omics data for accuracy using ML and DL algorithms. ML techniques, including Random Forest (RF), Naive Bayes (NB), Decision Tree (DT), Logistic Regression (LR), Gradient Boosting (GB), and DL methods such as Recurrent Neural Networks (RNN) and Feedforward Neural Networks (FNN) are compared. GB achieved 97 % accuracy in ML, while RNN outperformed by achieving 98 % accuracy in DL. This approach filters unclassified data effectively, demonstrating the significance of DL for leukemia prediction. The testing validation was based on 17 different features such as patient age, sex, mutation type, treatment methods, chromosomes, and others. Our study compares ML and DL techniques and chooses the best technique that gives optimum results. The study emphasizes the implications of high-throughput technology in healthcare, offering improved patient care.

## 1 Introduction

The multi-omics approach's integration has led to significant progress in the field of biomedical research nowadays. The word “multi-omics” expresses its central significance, implying a comprehensive investigation of biological systems through the simultaneous analysis of several biological informational tiers. System biology, tailored therapeutics, and holistic insights are some of the implications of multi-omics. Conventional biological research frequently focuses on just one molecular level, such as proteomics or genomes. Whereas, data analysis techniques and generation biotechnology have significantly advanced in multiple aspects. Multi-omics aims to integrate multiple omics such as genomes, transcriptomics, proteomics, metabolomics, and other such studies to understand the complex molecular interactions within cells. As a result of this recognition, testing and experiments based on multi-omics approaches have made breakthroughs lately in several medical science fields, such as skin disease, cancer research [1], microbiome analysis, and drug development. Hence the detailed research on multi-omics studies provides insight into complex biological systems and as a result, researchers are encouraged to find out the novel treatment targets, biomarker development and precision medicine. The healthcare experts realized that the combination of various omics datasets could lead to a more comprehensive understanding of biological processes. In the years to come, key research on multi-omics is expected to play a pivotal role in expanding our understanding of biotechnology and solving difficult healthcare issues such as cellular malfunctioning and sudden disease outbreaks.

**Fig. 1:**
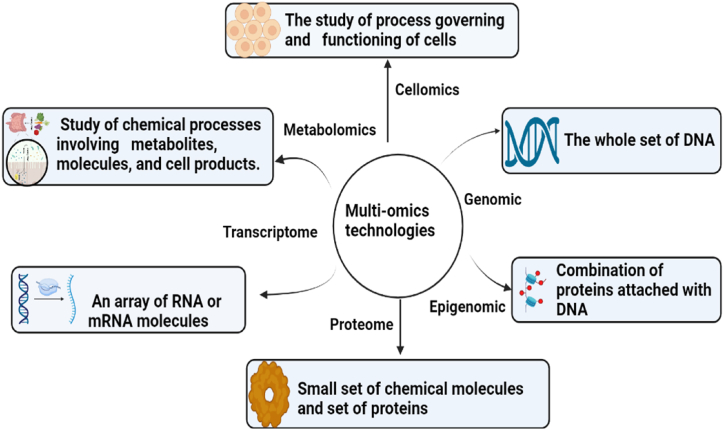
Overview of omics technologies.

The study of the relationships, their interaction and roles between various biological components, including proteomics, metabolomics, transcriptomics, genomes, and epigenomics, is the main goal of omics studies [2]. It allows scientists to understand how different layers of biological blueprints interact. Multi-omics studies involve a complex technique for biological analysis that focuses on heterogeneous datasets from various omics fields [3]. Personalized treatment options are developed more rapidly and disease diagnosis has reached an advanced stage by the ability of machine intelligence to integrate numerous biological datasets. Scientists now examine multiple omics datasets simultaneously to diagnose diseases at an early stage, to offer personalized treatment techniques and to identify potential root causes of cell malfunctioning. Multi-omics approaches present cutting-edge omics technology to assess biotechnical analytics and systems simultaneously [4]. Because cells form the basis of all living organisms, the importance of multi-omics is preeminent in unravelling biological complexities in the real world. A myriad of biological molecules builds them up, collaborating seamlessly to perform specific tasks. They are modelled as micro-automated factories with four main molecular components, namely Deoxyribonucleic acid, Ribonucleic acid, proteins, and metabolites [5]. The scientific fields studying these domains are called omics, and they all arise from high-throughput experimental strategies. These data types reveal the complexity of cells and their role in living organisms [6]. Fig. 1 demonstrates various multi-omics technologies used by researchers in healthcare and bioinformatics.

Diagnostic accuracy for diseases has improved recently. The approaches for cancer detection, prognosis, and therapy have been significantly enhanced by cutting-edge methods, including high-throughput sequencing, screening technologies, and artificial intelligence. This has led the medical domain to gradually shift towards precision medicine, achieved by combining the analysis of clinical and omics data [7]. The functioning and rules of the numerous relationships among omics technologies are part of biological processes [8]. Although it is not essential, it is preferred to segregate these levels to comprehend how they interact [9].

Several biological issues have been addressed using omics data, including tailored complicated disease therapy, medication development, and cancer therapeutic target identification [10,11]. However, drawing meaningful conclusions from complex, high-dimensional, and heterogeneous data can be difficult. Multi-omics data allows researchers to better comprehend biological systems through complementary functions [12]. To address this challenge, different Deep Learning (DL) and Machine Learning (ML) algorithms [13] have been gaining momentum in predicting diseases such as coronary artery disease [14,15] and COVID-19 [16] in recent years [17]. Both ML and DL are widely used in different areas, including medical sciences, Unmanned Aerial Vehicular Networks (UAV) [18], agriculture [[19], [20], [21]], fraud detection, network security and so on. ML is an artificial intelligence subset that uses statistical methods to analyze and improve predictions through data processing experiences. Many diseases can be predicted today using ML and DL algorithms. ML has made considerable strides in the previous several decades, developing complicated learning algorithms and effective pre-processing methods [22,23]. The development of Artificial Neural Networks (ANNs) into Deep Neural Network (DNN) topologies with gradually boosted DL learning capabilities was one of these breakthroughs [24,25].

The specialized branch of ML known as DL is the best option to simulate complex data processing mechanisms [26]. DL focuses on developing and utilizing intricate artificial neural networks with multiple interconnected layers that can extract hierarchical features from raw data. This allows DL algorithms to automatically learn and comprehend intricate patterns and representations, mimicking the first cognition and computation in the human brain inspired by biological models [27,28]. DL applications have revolutionized various domains of everyday activities like image and speech recognition, natural language processing, medical diagnostics, internet search, fraud detection, email/spam filtering, financial risk modelling, and more [29,30].

Although it can affect other blood types, leukemia most usually affects white blood cells [31,32]. Whether it shows acute leukemia with fast growth or chronic leukemia with slower growth, all forms of leukemia are primarily based on myeloid or lymphoid cells. Both adults and children are susceptible to leukemia [33,34]. According to earlier studies [35], Acute Myeloid Leukemia (AML) can be detected in patients under a range of Age 18–60 years with a survival rate of 2 years. Over the past three decades, children with Acute Myeloid Leukemia (AML) have experienced improved outcomes, about 70–75 % of overall survival and about 60–65 % of event-free survival. This upgrading is attributed to better supportive care and treatment optimization [36,37]. The 49-childhood cancer-type Acute Lymphoblastic Leukemia (ALL) cell lines were subjected to a multi-omics analysis, which quantified over 12,000 proteins and transcripts and showed sensitivity to 528 oncology and experimental medicines. Precision medicine can use this information to enable multi-omics phenotyping unique to a certain subtype [38].

To evaluate the effectiveness of ML systems, it is common practice to test programmed or instructed systems. However, when it comes to addressing hidden layers, DL is the go-to approach. DNNs, characterized by their complex neural structures, typically feature numerous hidden layers and advanced neurons, unlike traditional ANNs [39,40]. This means they can employ intricate procedures (e.g., convolutions) or multiple activations within a single neuron instead of simple activation functions [41,42].

**Fig. 2:**
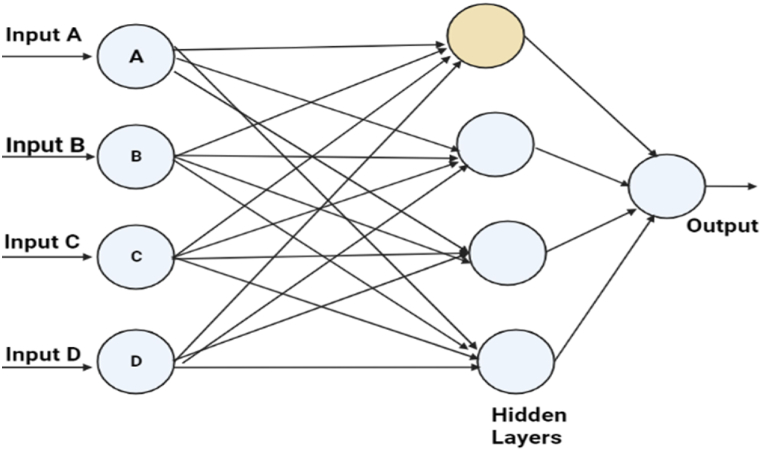
Example of artificial neural networks.

The DNN with several concealed layers as illustrated in Fig. 2, enables the connection of several perceptron units, allowing the input of one unit to serve as the output for another. Typically, there might be several secret layers [43]. The nodes of a layer may all be considered neurons. Assume that L stands for layers, *W*^1^, *W*^2^ for weights, and b1, b2 for bias. The bias used for both layers must be originally specified, and the weights must be declared randomly to prevent identical output from all units. The computations will be performed entirely from scratch and can be calculated using the following equations:

(1) $$L^{\left[1\right]}=W^{\left[1\right]}x+b^{\left[1\right]}$$

(2) $$a^{\left[1\right]i}=Activation\;Function\;\left(L^{\left[1\right]}\right)$$

(3) $$L^{\left[2\right]}=W^{\left[2\right]}a^{\left[1\right]}+b^{\left[2\right]}$$

(4) $$x=a^{\left[2\right]}=\delta \left(L^{\left[2\right]}\right)$$

(5) $$x_{predictions}=\left\{ \begin{array}{c} 1,if\;a^{\left[2\right]}>0.5\\ 0,Otherwise \end{array}\right.$$

Unlike traditional ML-based disease detection systems, we exploit both ML and DL with five ML classifiers and two DL classifiers. Moreover, we evaluated the performance of these classifiers in terms of accuracy in detecting disease. This study proposes a novel approach for integrating multi-omics by utilizing ML and DL techniques to predict leukemia. We experimented with our proposed research on a 3-genomics dataset and used features to select the correlation coefficient. This paper defines the eight phases for novel techniques based on ML and DL algorithms that use genomic technology data to predict leukemia. Our key investigation is as follows:

• Analyzing and experimenting on the 3-different genomic technology datasets with different techniques.
• Encoding the categorical values with the help of the encoding method.
• Refining the missing values with the help of the K-Nearest Neighbor (KNN) imputer.
• Select the features with the positive relationship of the correlation coefficient.
• Testing through 5 different ML algorithms, namely; Random Forest (RF), Naïve Bayes (NB), Gradient Boost (GB), Decision Tree (DT), and Reinforcement Learning (RL) to predict the disease with 17 different features.
• Applying Recurrent Neural Network (RNN) and Feedforward Neural Network (FNN) with Relu and Softmax Activation Function gives promising results.
• Selecting Binary cross_entropy and Mean_squared_error as loss functions to achieve good accuracy rates of neural networks.
• Presenting a comparison between ML and DL techniques with various feature selections such as age, Mutation type, chromosome, treatment type, affected, and many more.

The remainder of the paper is organized as follows. Section 2 describes the literature work studied in the era of multi-omics data integration and highlights its limitations. Section 3 presents the methods and materials discussed in this study, such as datasets, preprocessing, feature selection, and correlation coefficient. Section 4 highlights the main findings and discussions of the study. Finally, Section 5 describes the conclusion and future directions of the proposed research work.

## 2 Literature review

### 2.1 Deep learning technique

Deep learning-based Autoencoder optimizes model performance in extracting useful information. A variation Autoencoder model was presented in this study [44] to obtain attributes from multi-omics data of reduced dimensions and classify the samples. The model was evaluated with 450,000 RNA-seq gene expression and DNA methylation data. Their model was 97.41 % accurate and could classify 33 different kinds of cancers. The authors in the study [45] developed a deep learning framework called DCAP for cancer risk estimation, outperforming other methods by > 6.5 % C-index value. MRNA performed best, miRNA and methylation second and third, and copy number variation least. One of the most crucial tasks in such cases is choosing features to predict how well ML and DL algorithms will work.

Based on DNN, the MOLI method predicts drug response using somatic mutation, CNA, and gene expression data, resulting in more efficient results than single-omics and primitive integration multi-omics approaches. It is the first pan-drug approach for targeted drugs [46] The study [47] introduces the SALMON algorithm and Cox proportional hazards regression networks for breast cancer survival prognosis, generating prognostic biomarkers that integrate multi-omics using DL. In Refs. [47,48], the authors propose a subtype-GAN as a DL-based cancer subtyping approach for accurately characterizing multi-omics data, using input, common, and output layers for diverse distributions.

### 2.2 Machine learning technique

In reference [49], the authors summarized multiple representation learning approaches, with an emphasis on the Principal Component Analysis (PCA) and Marginal Fisher Analysis (MFA) methods. The authors used the k-means technique to evaluate the TCGA Breast Cancer and TARGET case studies. The Calinski-Harabasz average is used to evaluate clusters' new index. The genome is commonly employed for precise cancer therapy.

In reference [29], to distinguish AML samples from bone marrow cytomorphology, an ML method for cell segmentation and image categorization has been proposed. For binary classification models, the FRCNN achieved 0.97 AUC and 0.97 cell segmentation accuracy. In Ref. [50] data integration in a lung cancer study identifies joint and individual components from multiple omics data sources. This improves the prediction quality and classification of metastatic or non-metastatic cancer. The study validates prediction models using a 10-fold cross-validation framework and RFs. Integrative analysis outperforms non-integrative analysis for case-control status. Compared to supervised variable selection and lasso models, aJIVE models perform similarly for case vs. control and substantially better for metastasis.

The Boruta method for feature selection over the GDSC and CCLE datasets is used in this study [51] to predict drug cancer. The AutoBorutaRF, SVM-RFE, NB, Fselector, and autohidden approaches were considered for assessment. Results show that AutoBorutaRF reaches 71 %.

Most extra-cranial solid tumors in children are neuroblastomas, which are extremely prevalent. Gene expression data and CNA data were used to train the supervised classification algorithms, including XGBoost in this study [52] utilizing various machine-learning approaches. Support Vector Machine (SVM) was used with the GE dataset to get the maximum accuracy of 99 %. With both datasets, the Xgboost method performs well overall.

CFC significantly improved cancer driver gene identification performance using the deep forest to combine multi-omics data from 33 cancer types. It discovered 275 potential driver genes on the Pan-Cancer dataset, with 179 (65.09 %) in the CGC dataset. CFC identified 733 potential driver genes across 33 cancer types [53].

In another study [54], the author proposes the CANTARE method, a novel method for creating interpretable multi-omic models shown in an IBD case study. With features from more genomes, the system competes favourably with RFs and elastic net penalized regression. The best-performing model's biological implications are examined, along with the methodology's weaknesses and assets.

Most of the prior studies focused on DNNs for cancer prediction and tested traditional ML algorithms. The studies were often limited to using one of two DL or ML algorithms while different feature selection methods were utilized. ML and DL methods exhibit varying performance across datasets and tasks. Unlike traditional related work mentioned in the literature, our proposed work exploits novel techniques by integrating multi-omics for the prediction of Leukemia by utilizing ML and DL techniques on five different classifiers. Moreover, we evaluate the accuracy of ML and DL classifiers and propose the most accurate classifiers for detecting leukemia disease. Thus, our proposed research predicts the disease with high accuracy. To analyze complicated, high-dimensional, heterogeneous data and better capture nonlinearities and complex correlations in multi-omics data, DL is a rapidly evolving subset of ML to meet those challenges. Table 1 presents a comparative performance analysis of various classifiers on different datasets.

**Table 1:** Comparison of the proposed approach with previous work.

Work	Study Goal	Primary Data source	Sample Size	Model (ML& DL)	Type of Cancer	Outcome of Model
Proposed work	Propose Novel leukemia prediction techniques.	ICGC (2020)	10,095	RF, DT, NB, GB, LR, FNN and RNN	Leukemia	RNN = 98 % Feedforward = 97 % DT = GB = 97 % RF = LR = 96 % NB = 73 %
[55]	A three-stage DL system detects and classifies WBCs in bone marrow images	SCMC	1732	Convolutional neural network (CNN)	acute lymphoid leukemia	Accuracy = 89 %
[56]	Predicting patient prognosis statuses.	cBioPortal	1980	SVM, RF, DNN	breast cancer	SVM = 67 % RF = 72 % DNN = 71 %
[57]	Prediction of neuroblastoma survival using DNNss, machine learning	TARGET & SEQC	16,51	SVM, RF, LR, DT, DNN	Neuroblastoma	SVM = 60 % RF = 54 % DT = 48 % LR = 61 % DNN = 65 %
[58]	New multi-view feature selection algorithm based on CCA statistical method.	UCSC Xena functional genomics browser	25,57	RF, LR, XGB	kidney renal clear cell carcinoma	RF = 59 % LR = 69 % XGB = 76 %
[59]	Proposed Multi-view Factorization Au-toEncoder for integrating multi-omic data with domain knowledge.	TCGA-BLCA, TCGA-LGG	10,546	SVM, AdaBoost, DT, NB, RF, Variational Autoencoder (VAE), Adversarial Autoencoder (AAE)	Cancer	SVM = 68 % DT = 57 % NB = 63 % RF = 67 % VAE = 56 % AAE = 69 %
[1]	Benign and Malignant Skin Disease Prediction Using ML	ISIC	3297, 1649, 825, and 210 images	KNN	Skin cancer	79.24 %, 79.39 %, 83.63 %, and 100 % for, respectively 3297, 1649, 825, and 210

In Table 1, our research unfolds an innovative approach that harnesses the power of both ML and DL classifiers to tackle the intricate challenge of diagnosing leukemia diseases. Notably, our proposed classifiers exhibit significantly superior accuracy levels compared to the previously mentioned studies in the healthcare domain. Table 2 illustrates notations and their description.

**Table 2:** Summary of notations.

**Notation**	**Description**
L	Layers
W	Weight
b	Bias
a	Activation Function
H	Samples
T_m_	The measure of Variable 1
S	The measure of variable 2
Σ	Summation
A	Relu Activation Function
Y	Variable
P	Softmax Function
*Z*_*i*_	Neurons in the output layer’s value
Exp	Exponential Function
Loss	The variable that stores the value of the Loss Function
X	Name of label
Ce	A score calculated for cross_entropy
N	Number of classes
*y*_*i*_	True values for that class
*p*_*i*_	Predicted values for that class
Log	Calculate the log value for the predicted class
SE	Variable for storing the value of mean_squarred_error
G	Total number of samples

## 3 Material and methods

### 3.1 Datasets collections

To perform ML and DL classification, we initially collected a dataset from a publicly available source ICGC Data portal [60]. The datasets have three categories of multi-omics data: Donor, Simple Somatic Mutation, and Specimen. The donor file includes information on the patient's history, such as age, gender, length of survival, and donor relapse type. 5983 samples total are included in the simple somatic mutation, all of which have been examined and approved by the Illumina HiSeq verification platform. There are 2, 844 samples in the specimen file. Table 3 illustrates the complete details of the dataset.

**Table 3:** Dataset samples.

Classes	Records per class	Features
**Donor**	1267	10
**Simple Somatic Mutation**	5983	11
**Specimen**	2, 844	9

### 3.2 Pre-processing

We removed unreliable noisy data using preprocessing methods. Features with a missing score of more than 50 % and less than 20 % were excluded. Only features with more than 20 % and less than 50 % of the data missing score were included [61]. Missing data were imputed using the KNNimputer technique, which applies the k-neighbor algorithms criteria [62]. To balance the dataset, this study exploited a SMOTE technique [63], which balanced the dataset sized to 1690 out of which 1436 samples (85 %) were assigned to training and 254 samples (15 %) were assigned to testing. Moreover, no SMOTE technique was utilized in DL classification, which contains 1267 samples. A well-known high-level Python programming was used [64]. Table 4 depicts the division of the dataset by our pre-processed dataset.

**Table 4:** Division of training and testing samples.

ML		DL		
Training	Testing	Training	Validation	Testing
**(1436, 17)**	(254, 17)	(633, 17)	(317, 17)	(317, 17)

#### 3.2.1 Feature selection

**Fig. 3:**
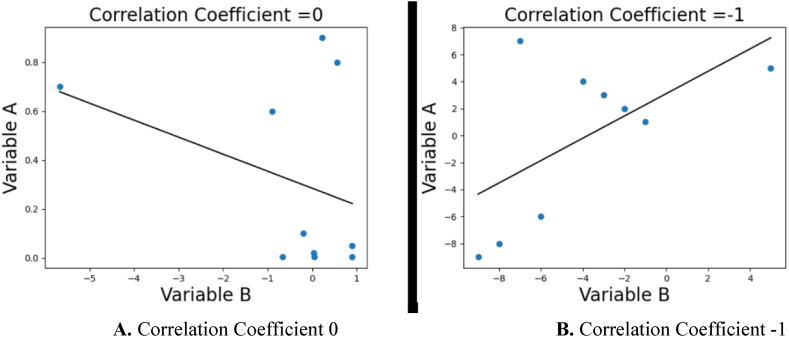
**A.** Correlation coefficient 0 **B.** Correlation coefficient −1.

A correlation is associated with the relationship between the two variables, and multi-variable and bivariate statistics are possible [65,66]. Consider two variables with the labels A and B. The relationship among variables can be measured with values that fall from −1 to 1. To assists the understanding of correlation among variable, the value or coefficient is scaled within this range [67]. If the value of the coefficient is 0, that implies there is no link between variables A and B. Conversely, if the coefficient is between −1 and 1, either A to B or B to A is precisely predicted (see Fig. 3). Later in the twenty-first century, one of the key techniques in epidemiological research is correlational analysis [68]. In this study, Pearson’s linear correlation was estimated [69]. Correlation may be displayed in many ways, like graphs, matrices, and other similar tools. It is possible to define Pearson's mathematical equation as:

(6) $$p=\frac{H\left(\Sigma ts\right)-\left(\Sigma t\right).\left(\Sigma s\right)}{\sqrt{H\left(\Sigma t^{2}\right)-{\left(\Sigma s\right)}^{2}}\sqrt{n\left(\Sigma s^{2}\right)-{\left(\Sigma s\right)}^{2}}}$$

where, *H* denotes the samples, *t* denotes the measures for variable 1, *s* denotes the measure for variable 2, Σts denotes the sum of the product of variable *ts*, Σt denotes the sum of variable *t’s* measures, Σs denotes the sum of variable *s*’s measures, $\Sigma t^{2}$ denotes variable *t’s* square and its sum, $\Sigma s^{2}$ denotes the variables *s*’s square then its sum.

Fig. 3A and B depict the coefficient ranges, shedding light on the intricate relationships between variables A and B. In this connection, Fig. 3A shows a coefficient value of 0, signifying the absence of any discernible relationship between A and B variables. On the other hand, Fig. 3B presents a compelling narrative with a coefficient value of −1, unequivocally indicating a robust negative relationship among the variables under examination. This visual information increases our understanding of the intricate interplay between these factors and underscores the significance of the findings within the broader scope of the research.

**Fig. 4:**
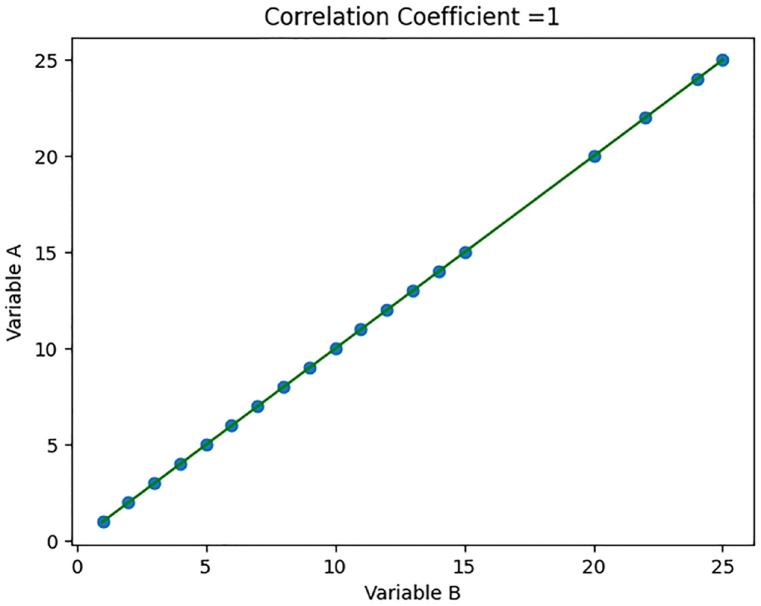
Correlation Coefficient with a positive relation.

In Fig. 4, the coefficient value stands at an impressive 1. This underscores a highly significant and positively correlated relationship between the variables under examination. The data presented in this figure leaves no room for doubt, clearly demonstrating the strength and directionality of this association. Such a strong correlation, as depicted, has important implications for our understanding of the phenomenon being studied. This compelling visual insight highlights the key findings of our analysis.

### 3.3 Proposed novel technique

Different from traditional research for detecting and predicting leukemia disease, our proposed research exploits both ML and DL. The proposed research includes the latest techniques to predict leukemia disease using various ML and DL classifiers, including RF, DT, LR, GB, NB, FNN, and RNN [[70], [71], [72]]. After selecting the most relevant features, we obtained 10,095 samples. We determined the best characteristics from the dataset using the correlation coefficient. We evaluated our approach using the Receiver Operating Characteristic (ROC) curve, confusion matrix, and accuracy rate. Algorithm-1 outlines the ML algorithm process, while Algorithm-2 describes how the DL algorithms work.

**Fig. 5:**
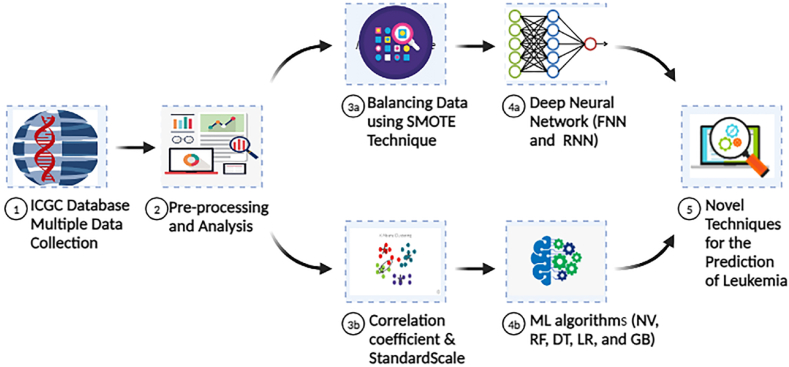
Proposed methodology.

In alignment with the outlined methodology, Fig. 5 illustrates the proposed approach. This figure is positioned below to visually represent the workflow described in this section.

**Fig. 6:**
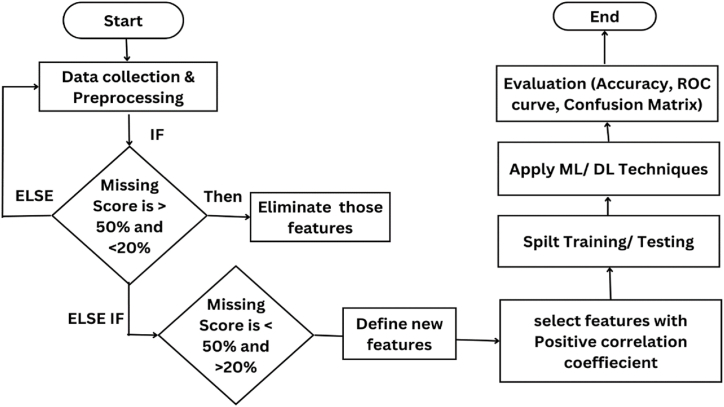
Flow chart of research study.

Our study's flow of steps is depicted in Fig. 6. During preprocessing, our algorithm will eliminate features with a missing score greater than 50 % but less than 20 %, while retaining only those with a missing score less than 50 % and greater than 20 % in a new dataset.

## 4 Experimental results

To introduce a novel technique for leukemia disease forecasting, this study utilized two distinct methods, an ML-based approach and a DNN technique, with experiments conducted using Python programming language on Windows 10 with a 1.80 GHZ CPU. A diverse range of ML and DL algorithms were employed to predict the occurrence of leukemia disease by incorporating different sets of features.

### 4.1 Machine learning techniques

**Fig. 7:**
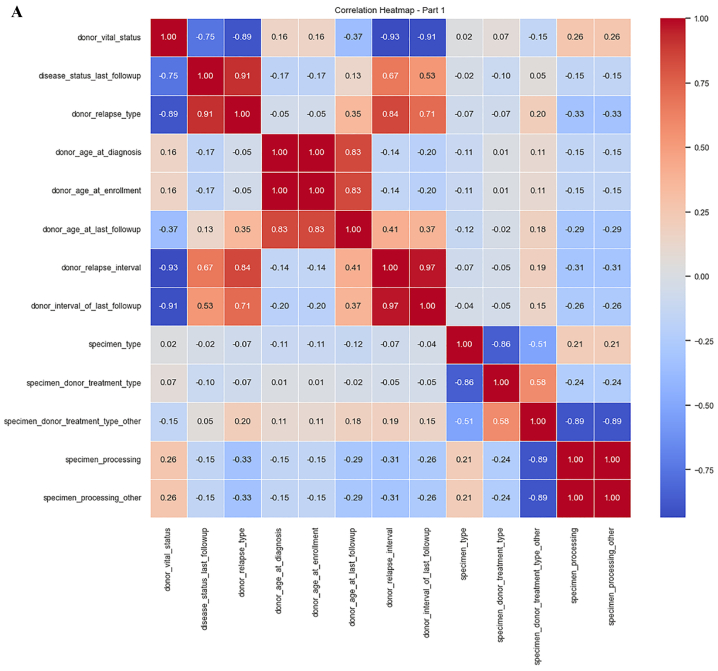

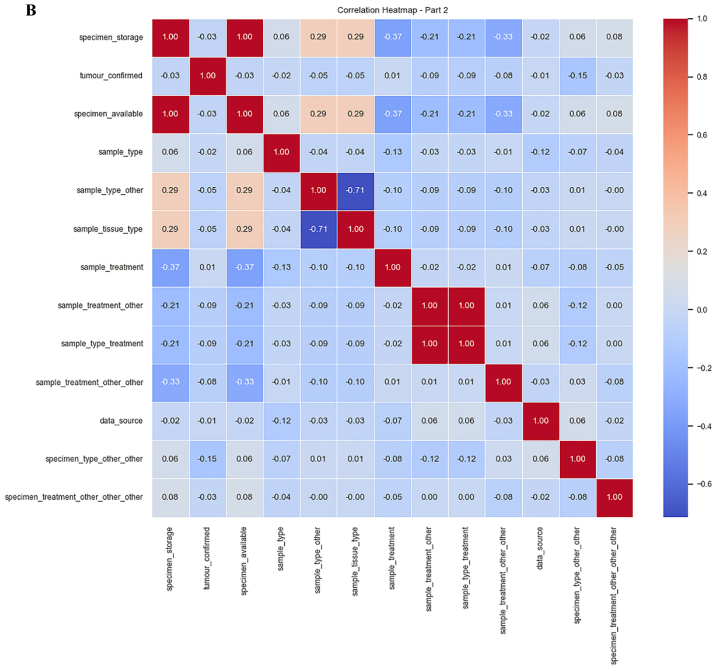
**A.** Correlation coefficient (part 1). **B.** Correlation coefficient (part 2).

We used a correlation coefficient to select the best features. The correlation matrix in tabular form is shown in Fig. 7A and B. These figures represent the correlation matrix between various features. The value at the diagonal of the matrix is 1 means that each feature is correlated with itself perfectly. When we get the correlation coefficient value of 1, it means there is a positive relationship among features, and in our study, most features have a positive correlation.

**Fig. 8:**
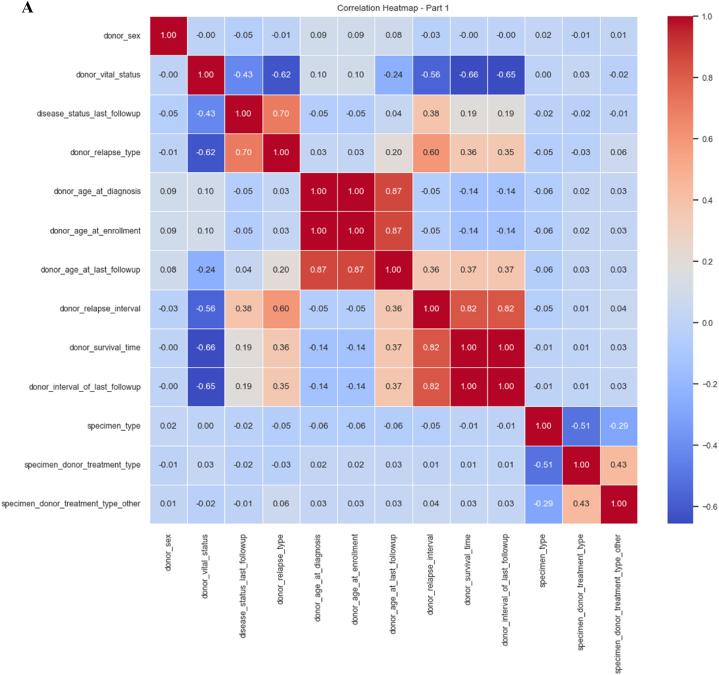

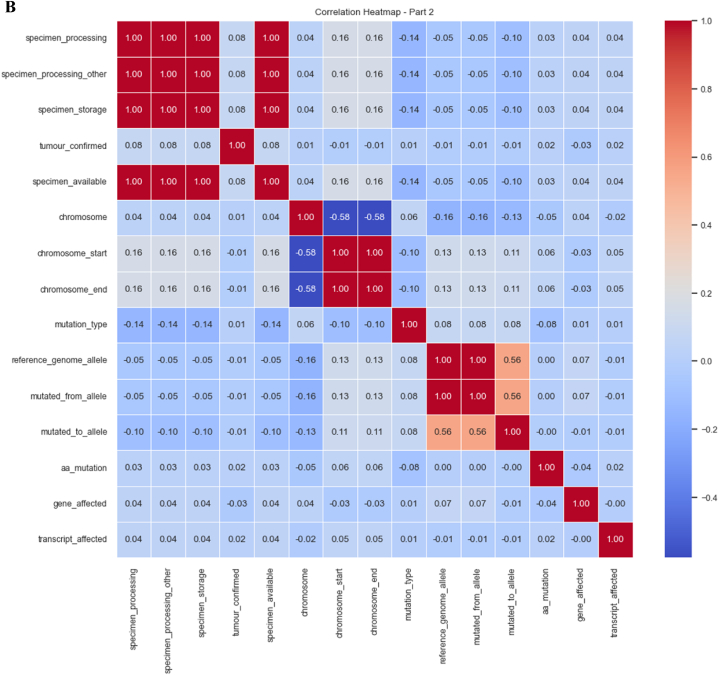

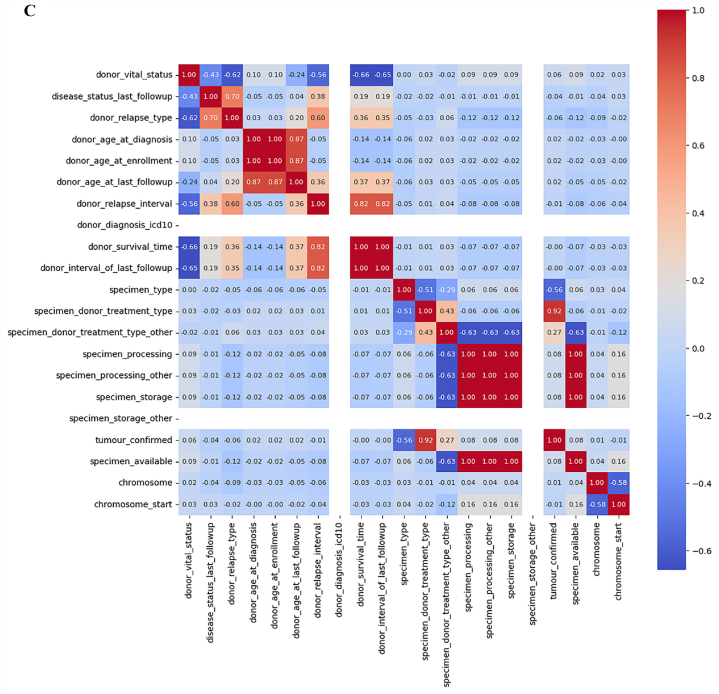
**A.** Consolidated features (part 1). **B.** Consolidated features (part 2). **C.** consolidated features (part 3).

Fig. 8A, B and C depict consolidated features for our analysis. We can observe that the coefficient value for most of the features ranges between 0 and 1. Very small features have negative coefficient values. The values that fall under the negative range have less importance as compared to those between 0 and 1. We consider the positive values for further investigation.

Table 5 exhibits the implementation of the various algorithms with imbalanced data after applying SMOTE techniques for balancing the dataset. We can see a difference between the algorithm's performances with imbalanced data and balanced data. As we can see, the performance of algorithms before and after sampling varied significantly. With imbalanced data, almost all algorithms predict 100 % performance, while with balanced data, all algorithms predict accurately. The overall performance of DT and GB algorithms was observed suitable. At the same time, the NB performs at an average.

**Table 5:** Performance of ML algorithms with imbalanced data.

ML Algorithms	Imbalanced data				Balanced Dataset			
	Accuracy	Precision	Recall	F1	Accuracy	Precision	Recall	F1
**Logistic Regression**	100 %	99 %	100 %	100 %	96 %	94 %	95 %	96 %
**Random Forest**	100 %	100 %	100 %	95 %	96 %	94 %	96 %	96 %
**Decision Tree**	100 %	99 %	99 %	100 %	97 %	95 %	97 %	96 %
**Gradient Boosting**	100 %	100 %	98 %	100 %	97 %	96 %	98 %	97 %
**Naïve Bayes**	99 %	98 %	100 %	73 %	73 %	68 %	62 %	65 %

**Fig. 9:**
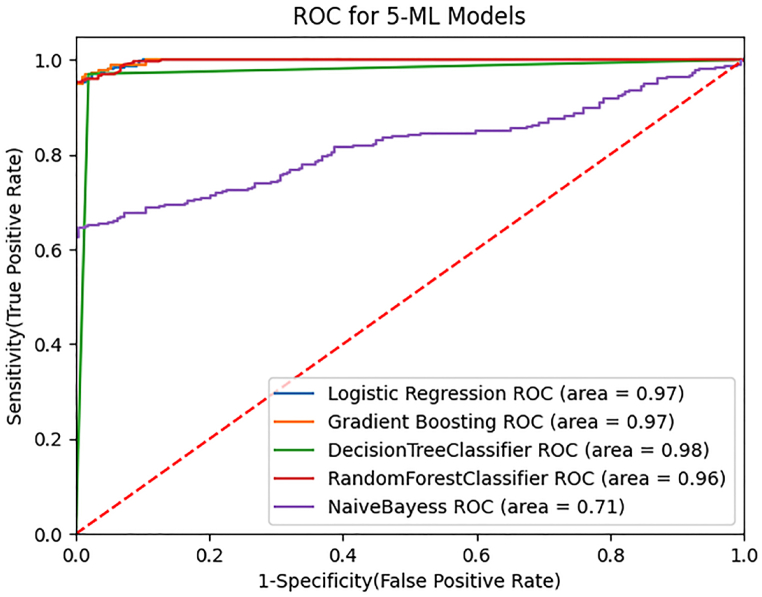
Combined ROC curves for classification models.

The ROC curves can be used for visualizing the performance of classification models. This is a graphical illustration of a quantitative data model's performance for various test values, with its sensitivity (percentage of true positives) against the proportion of false positives (1-specificity). See Fig. 9 below:

Fig. 9 describes the ROC curves in terms of specificity (False Positive Rate) and sensitivity (True Positive Rate). If the curve is turned to the upper left corner, the model can be classified and perform well. The five classification techniques may be seen slanting to the upper left, showing vital algorithm accuracy, whereas DT achieved the highest accuracy (98 %) and both LR and GB illustrated (97 %) accuracy. Similarly, the RF classifier has also achieved satisfactory accuracy (96 %). Conversely, NB yielded poor accuracy (71 %). Thus, DT can be considered the best ML classifier.

**Fig. 10:**
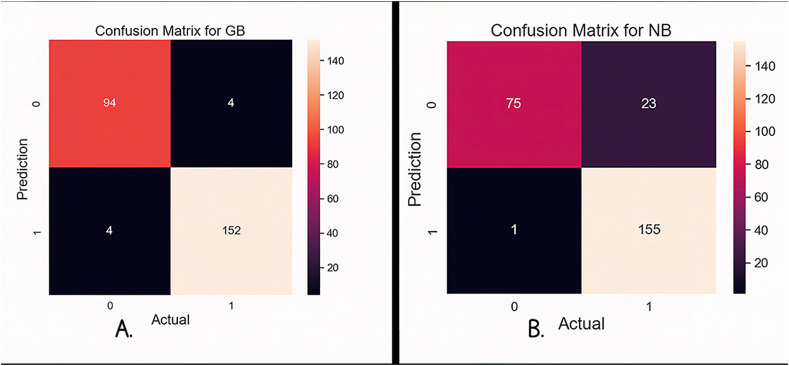
**A.** Confusion matrix for GB; **B.** Confusion matrix for NB.

In Fig. 10A, we present the confusion matrix for the GB algorithm, representing its robust accuracy in accurately predicting 246 out of 254 samples, with only 8 samples being predicted inaccurately. On the other hand, in Fig. 10B, we provide the confusion matrix for the NB algorithm. It demonstrates the accurate predictions of 230 samples out of the 254 samples, with only 24 being incorrectly predicted. These visual representations witness the algorithmic performance and provide valuable insights into their predictive capabilities.

**Fig. 11:**
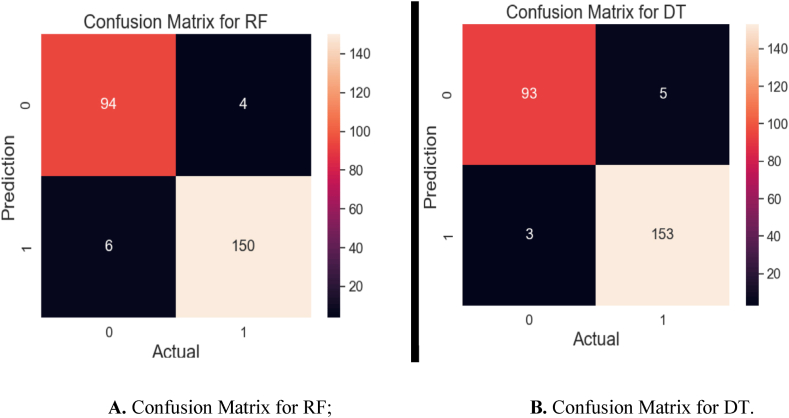
**A.** Confusion matrix for RF; **B.** Confusion matrix for DT.

Fig. 11A presents the confusion matrix for the RF algorithm, which was evaluated on 254 test samples. Remarkably, the model's accuracy was high as it accurately predicted 244 of these samples, with just 10 cases of misclassification. The confusion matrix for the DT algorithm is based on 254 test samples as shown in Fig. 11B. With only 8 samples misclassified, the DT model presents a good prediction ability by correctly categorizing 246 of these samples. The performance of the algorithms is demonstrated by the above visuals.

**Fig. 12:**
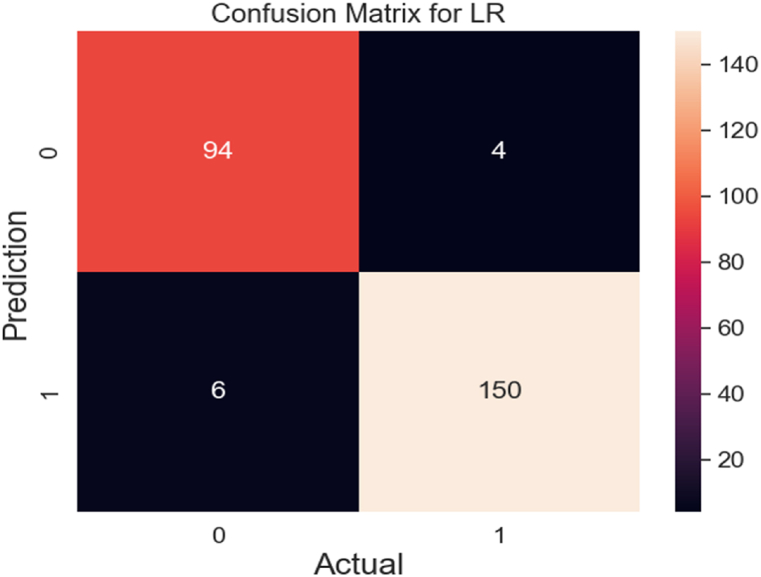
Confusion matrix for LR.

In Fig. 12, the confusion matrix for the LR method is shown which includes a total of 254 test samples. With only 10 data misclassified, the LR model has achieved impressively strong performance by correctly predicting 244 out of 254 samples. This degree of accuracy presents the algorithm's strong prediction capabilities, which are crucial for the proposed model. By excelling in its predictive accuracy, the LR algorithm contributes invaluable insights to our understanding of the dataset and its underlying pattern.

In summary, the GB algorithm emerges as the standout performer, consistently delivering precise predictions compared to other algorithms. Meanwhile, the NB algorithm appears to perform more moderately, indicating an average level of predictive accuracy. We can conclude that the GB algorithm is more effective in prediction than other algorithms, whereas NB has yielded average performance.

### 4.2 DL techniques

We train FNN [73,74] and RNN [75] with a sequential model that allows for layer-by-layer training [76]. The activation functions are an integral part of neural networks. Without activation functions, the neural network can be a simple linear regression model. A Rectified linear unit is [77,78] an activation function, which works with a threshold value of 0 and can be defined with the equation below:

(7) $$A\left(y\right)=\left\{ \begin{array}{c} \;0\;if\;y<0\\ \;y\;if\;y\geq 0 \end{array}\right.$$

where A defines the relu activation function, y denotes the variable. From the above equation, we can say that there are two possibilities for calculating the Relu of any variable. When the threshold value is 0, it produces 0; when the threshold value is more than 0, it creates a linear function. In addition, another function used in this study is the softmax function. The relative probabilities are computed using the Softmax activation function. Let's examine the working of the softmax function. The softmax returns the probabilities of every class. It can be calculated from the given below.

(8) $$p\left(Z_{i}\right)=\frac{\exp\left(Z_{i}\right)}{\Sigma \;\exp\left(Z_{i}\right)}$$

where P denotes the softmax function and *Z*_*i*_ represents the output layer’s value in the neurons.

The non-linear function in the form of exponential is defined. To normalize and convert them into probabilities, these values are divided by the sum of exponential values.

The batch sizes of 32 and Epoch 200 were tested. The convergence of learning to prevent overfitting was assessed using early halting criteria. Training neural network loss functions are essential [79]. Depending on the dataset's characteristics, the optimum loss functions must be chosen to achieve the best results. The loss function can be used to control how the model predicts outcomes. It will be a minor loss. If the model's predictions are more closely aligned with the actual data, the loss will be less; otherwise, it will be maximal [80]. Mathematically, it can be expressed as:

(9) $$Loss=t\left(x_{pred}-x_{actual}\right)$$

here, t is the model's name and *x* is the label.

We have used (BCE) and Mean Squared Error (MSE) as loss functions for our analysis [81]. BCE is the default loss function for situations involving binary classification. BCE compares the 2-2 probability distribution set over the same underlying set. BCE will calculate a score for the prediction that indicates the typical difference between the actual and projected probability distributions. BCE is equal to zero when the score is reduced [82]. The following equation can compute BCE.

(10) $$Ce=\sum_{i}^{n}y_{i}\;\log\;p_{i}$$

where *Ce* denotes the score to be calculated for cross-entropy. In this equation, n denotes the number of classes, *y*_*i*_ denotes the true values for that class, and *p*_*i*_ denotes the predicted values of the class. Calculate the log of predicted values *p*_*i*_. By using an Adams Optimizer to reduce BCE loss function as well as MSE, the model’s weights and biases were learned [81].

A typical metric for assessing a model's predictive performance is a Mean Squared Error (MSE), which can be calculated by:

(11) $$SE=\left(\frac{1}{G}\right)*\;\Sigma {\left(actual\;-prediction\right)}^{2}$$

here, Σ defines the sum of the square of the difference between of actual and predicted values, and *G* denotes the total number of samples.

Table 6 demonstrates the performance of FNN and RNN techniques. The models were trained with two different activation functions, and the performance of the model was calculated in terms of accuracy and two different types of loss functions, namely, MSE and BCE. There is an inverse relationship found between the loss and accuracy. Here, the real function with both activation functions provides exemplary accuracy. However, the accuracy of the relu function is much better than softmax. As compared to algorithms, RNNs perform better than FNNs. Since there is a lower value of MSE, the model can predict better. The lowest MSE obtained with actual function is 2.04 % and 3.77 %. The RNN algorithm achieved the highest BCE loss with the softmax activation function, which is 11.2 %. The lowest accuracy recorded is 57 %, obtained by FNN with a softmax activation function.

**Table 6:** Performance for DNN techniques.

DNN Technique	Softmax		Relu		Softmax		Relu	
	Mean Squared Error	Accuracy	Mean Squared Error	Accuracy	Binary Cross Entropy	Accuracy	Binary Cross Entropy	Accuracy
**FNN**	42 %	57 %	2.04 %	97 %	5.21 %	59 %	10.7 %	97 %
**RNN**	18.2 %	96 %	3.77 %	95 %	11.22 %	98 %	5.99 %	96 %

**Fig. 13:**
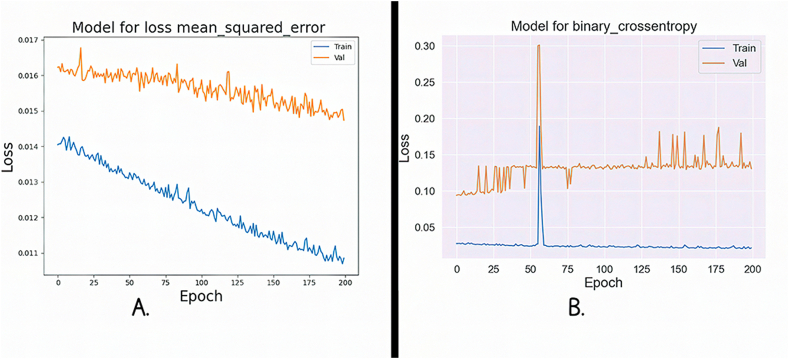
**A.** Mse loss for FNN; **B.** Bce loss for FNN.

Fig. 13A and B illustrate the training and validation loss curves for the FNN algorithm, using two distinct loss functions. The training and validation processes were conducted over 200 epochs, involving forward and backpropagation through the network layers. Fig. 13A shows MSE loss. Remarkably, both the training and validation losses gradually decrease as the number of epochs increases. Notably, the validation loss consistently remains lower than the training loss, suggesting that the model's generalization ability improves over time. In Fig. 13B, the BCE loss is depicted. Here, we observe an intriguing pattern: the training loss shows a linear increase with each epoch, while the validation loss moves towards the upper left corner of the plot. This tendency suggests that, despite the seemingly rising training loss, the model gradually improves its predictions and gets closer to producing precise and accurate outcomes.

**Fig. 14:**
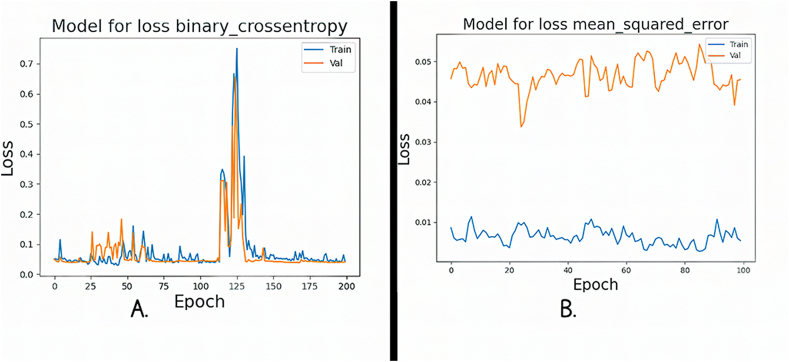
**A.** Bce loss for RNN; **B.** Mse loss for RNN.

The BCE loss function for RNN is shown in Fig. 14A, which shows how the training and validation losses follow a pattern of steady increase and decline as the number of epochs increases. However, there is a noticeable divergence as we approach 200 cycles, the loss rapidly increases, indicating a change in the training procedure. We now exhibit the MSE loss curve for the RNN in Fig. 14B. A continuous convergence of the model during training is indicated by the evaluation's test and training losses, which both show a smooth, gradual fluctuation. This consistency suggests that there are no sudden deviations or increases in loss, indicating that the RNN is successfully learning and generalizing from the input.

**Fig. 15:**
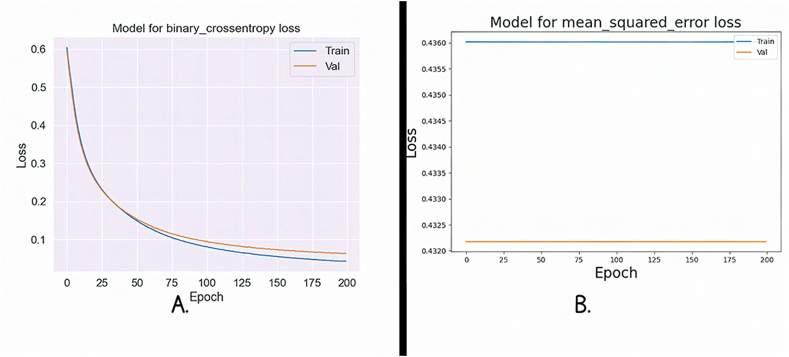
**A.** Bce loss for FNN; **B.** Mse loss for FNN.

Using two different loss functions, Fig. 15A and B present a clear examination of the loss aspects for the training and validation stages. In Fig. 15A, we concentrate on the BCE loss for the FNN. Interestingly, the training and validation losses show a striking closeness, nearly overlapping, as the number of epochs grows. This convergence suggests that FNN is effectively learning and generalizing from the data, as both training and validation experience a parallel decrease in loss. Fig. 15B, on the other hand, shows MSE loss for the same FNN. Here, a distinctive pattern emerges. The training loss follows a consistent, linear increase, resembling a straight line at the upper end of the graph. In contrast, the validation loss exhibits a parallel linear increase but at the opposite end, forming a straight line at the lower portion of the plot. This inconsistency in loss behavior between training and validation underscores the network's ability to adapt and potentially highlights areas where further fine-tuning may be necessary to enhance its performance.

**Fig. 16:**
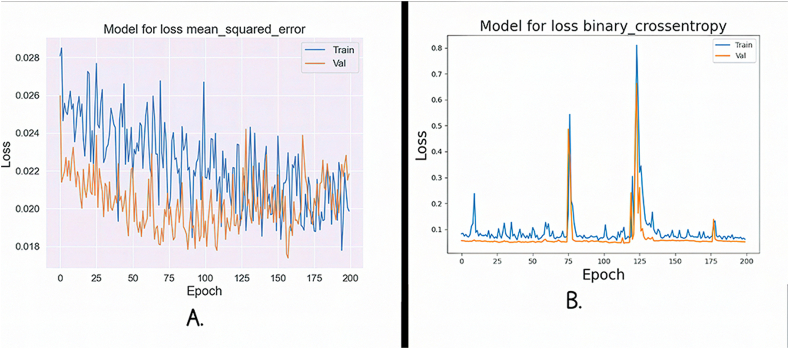
**A.** Mse loss for RNN; **B.** Bce loss for RNN.

In Fig. 16A, we provide the MSE loss function for the RNN. It is proved that as the number of epochs increases, the training and validation losses follow a smooth, gradual pattern of increase and decrease. Notably, training and validation start from nearly identical loss values, suggesting that the model's initial training had a solid foundation. This parallel behavior in loss signifies that the RNN is effectively learning and generalizing from the data, with a consistent trend towards convergence. In Fig. 16B, the highlighted BCE loss for the same RNN. Here, a distinctive pattern emerges. The training and validation losses overlap closely and initiate with an exceptionally high loss value. As training progresses, both losses slowly increase, but a notable deviation occurs between Epochs 75 to 100, where they rapidly reach their maximum loss values before declining. This loss during that period could indicate a critical point in the training process, potentially signaling the need for further investigation or adjustments in the model's architecture or training parameters.

**Fig. 17:**
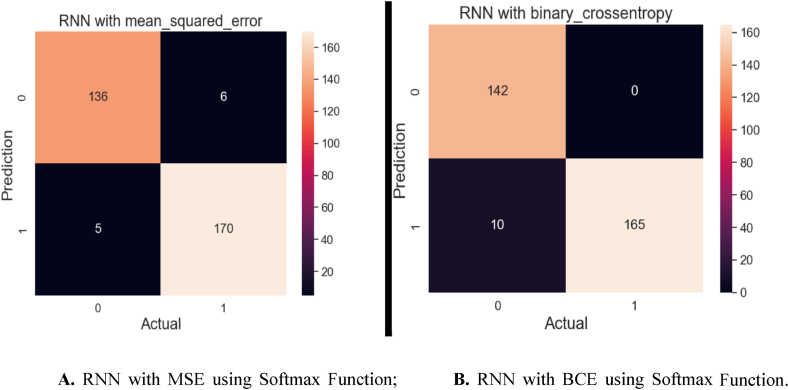
**A.** RNN with MSE using Softmax Function; **B.** RNN with BCE using Softmax Function.

Fig. 17A and B visualize a confusion matrix achieved from the RNN algorithms that employ two different loss functions: MSE and BCE. These models also utilized the softmax activation function for their predictions. Fig. 17A illustrates the confusion matrix for the RNN algorithm utilizing MSE as its loss function. This algorithm achieved a high accuracy, accurately predicting 306 out of 317 samples. However, it misclassified 11 samples. In Fig. 17B, we exhibit the confusion matrix for the RNN algorithm employed for BCE as its loss function. This model also exhibited a strong performance, accurately predicting 307 out of 317 samples. It showed 10 inaccurate predictions only. These confusion matrices provide valuable insights into the classification performance of the RNN algorithms using different loss functions. Both models exhibit a high level of accuracy, but the choice of loss function may impact the model's predictive behavior and ability to handle specific data characteristics. Further analysis and consideration of the specific problem domain may guide the selection of the most suitable loss function for future model training.

**Fig. 18:**
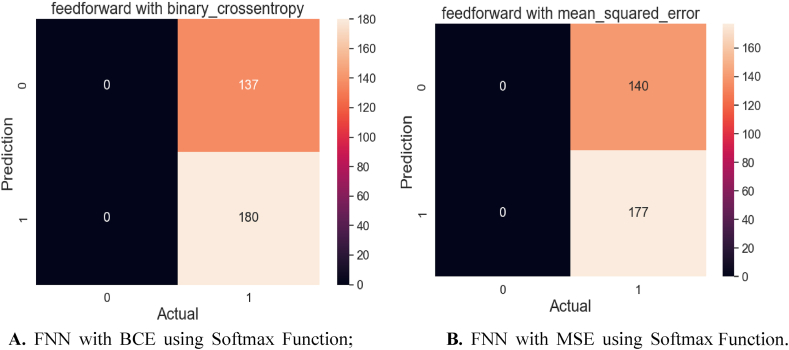
**A.** FNN with BCE using Softmax Function; **B.** FNN with MSE using Softmax Function.

Fig. 18A and B demonstrate the confusion matrix for the FNN algorithm with MSE and BCE loss functions. Fig. 18A depicts the confusion matrix for FNN with BCE loss. There are 317 samples in total in the test. The model accurately predicted 180 out of 317 samples, while the model incorrectly predicted 137 samples. Fig. 18B shows the confusion matrix for the FNN algorithm with MSE. The test has 317 samples in total. The model successfully predicted 177 out of 317 samples, whereas 140 samples were wrongly predicted.

**Fig. 19:**
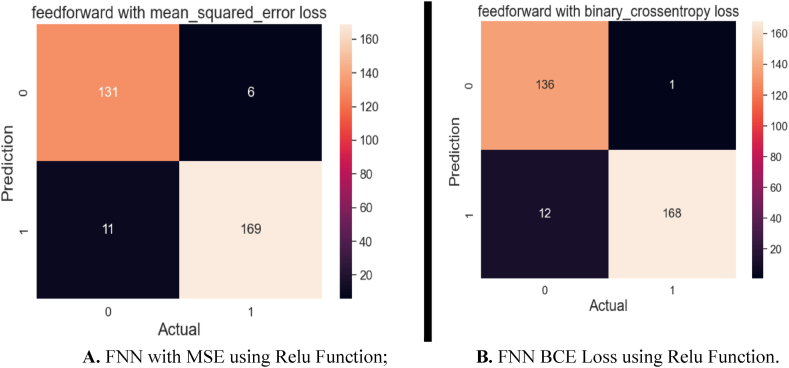
**A.** FNN with MSE using Relu Function; **B.** FNN BCE Loss using Relu Function.

Fig. 19A and B explain the confusion matrix for FNN with two loss functions: BCE and MSE. The relu activation function was applied. Fig. 19A shows the confusion matrix for the FNN algorithm with MSE Loss. The test has 317 samples in total. Out of 317 samples, the model successfully predicted 300, while 17 were predicted incorrectly. The confusion matrix for the FNN method with BCE loss is shown in Fig. 19B. The test comprises a total of 317 samples. The model correctly predicts 304 out of 317 samples, whereas 13 samples are wrongly predicted.

**Fig. 20:**
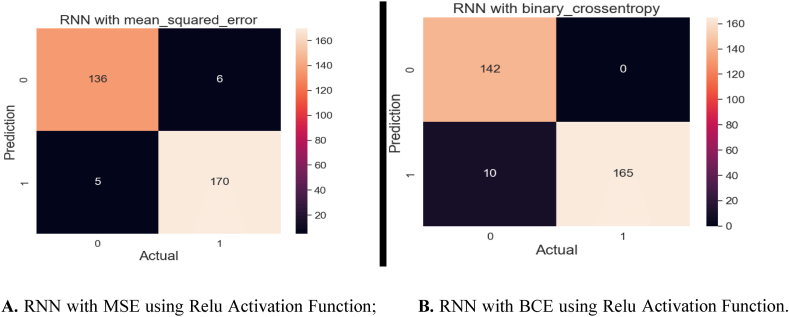
**A.** RNN with MSE using Relu Activation Function; **B.** RNN with BCE using Relu Activation Function.

The confusion matrix Fig. 20A for the RNN algorithm employs the MSE loss function. Among the 317 samples, this algorithm correctly predicted 306 of them, demonstrating its ability to classify the majority of the data accurately. However, it inaccurately predicted 11 samples, indicating some level of misclassification. Fig. 20B illustrates the results of the same algorithm but with a different loss function of BCE. Here, the algorithm accurately predicted 307 out of 317 samples, indicating a robust predictive performance. It yielded 10 incorrect predictions, demonstrating high precision and robustness in its classification capabilities. These confusion matrices provide a detailed breakdown of the model's classification performance, highlighting its accuracy and error rates. Such insights are invaluable for assessing the algorithm's effectiveness in handling the specific dataset and may guide further refinements in the model or data preprocessing if needed.

### 4.3 Comparative analysis

**Fig. 21:**
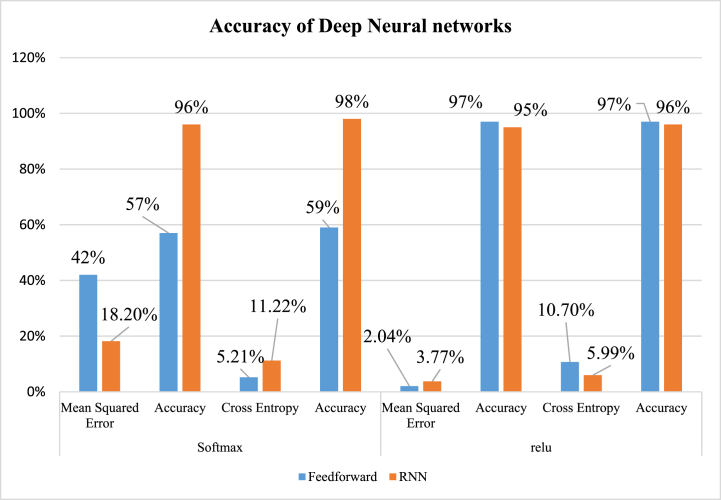
Overall performance of DNNs.

The purpose of the research is to apply several ML and DL algorithms to propose novel techniques for leukemia prediction. The accuracy rate of the performance is then compared here:

Fig. 21 represents the overall performance of DL techniques in terms of the loss function and activation function. Two DL techniques, FNN and RNN algorithms, were used in this study. A trivial difference is found in the accuracy of algorithms with Softmax and Relu functions. The highest accuracy was recorded, 98 % attained by RNN with low BCE loss, and the lowest accuracy was recorded, 57 % by the FNN algorithm.

**Fig. 22:**
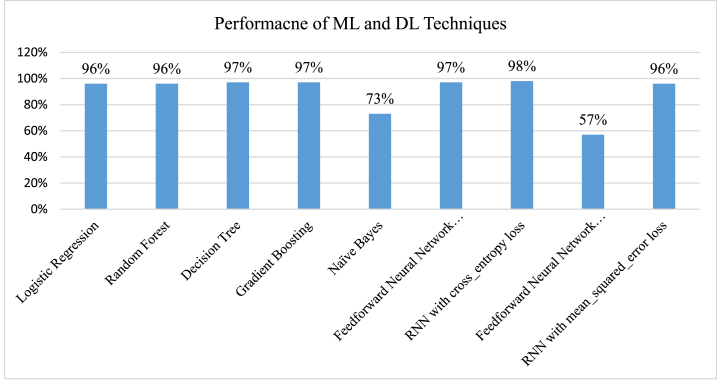
Comparison of ML and DL techniques.

Fig. 22 compares the performance of the ML and DL algorithms. Only NB performs at an average level among the 5-ML and 2-DL algorithms utilized in this study, with 4 ML algorithms achieving the greatest accuracy of 97 %. Both FNN and RNN algorithms with sequential models underwent training to determine the model's accuracy and loss function. The accuracy rate for the RNN method with BCE was 98 %, whereas the same algorithm with MSE was 96 %. We can see that the accuracy rates of ML and DL algorithms differ somewhat.

### 4.4 Discussions

Previous research has utilized machine learning (ML) and deep learning (DL) to predict leukemia cancer using single data [83,84]. To analyze data related to breast cancer and other types, prior investigations have preferred DL algorithms such as CNN, RNN, ANN, and VAE (Variational Autoencoder) with relu activation function and BSE as loss function [[85], [86], [87], [88], [89]]. Feature selection methods such as PCA, RF Recursive selection, and Chi-square have been widely used in earlier research. The analysis was mainly conducted using R and Python. Some of the common traditional ML algorithms are preferred in the studies, such as RF, SVM, DT, and Xgb [[90], [91], [92], [93]]. We have compared our work with previous studies, which are summarized in Table 1 above. Due to feature dependency, conventional machine learning algorithms have limitations, but deep learning's superior learning capability supports complicated biological research and advances genome-based disease prediction. In our work, we use ML and DL-based approaches to present a unique method for predicting leukemia. From the ICGC database, three separate omics datasets were retrieved. Features were chosen using the correlation coefficient, and SMOTE techniques were used to balance. The algorithms RF, DT, GB, LR, and NB were chosen for the predictions. While other 4-algorithms do well, only NB performs on average with them. A 97 % accuracy rate was the highest. The highest Precision, Recall, and f1 score attained by GB is 96 %, 98 % & 97 % respectively. The sequential model, Epoch 200, and batch size 32 were used to select and train the FNN and RNN neural networks. We utilized two loss functions (BCE and MSE) to evaluate DNNs and two activation functions (Relu and SoftMax). The RNN neural network outperformed the others with a 98 % accuracy rate. Both activation functions resulted in reasonable accuracy rates. Our study stands out from others due to its superior disease prediction accuracy, as evidenced by our comparison of various AI techniques discussed in the paper.

## 5 Conclusions and future work

This study presents a novel approach to predicting leukemia disease using ML and DL techniques. The 3-omics dataset was retrieved from the ICGC data portal and a correlation coefficient was applied to select the features. The SMOTE method was used to balance the data, and five well-known ML algorithms (RF, DT, NB, LR, and GB) were employed. RNN and FNN were also chosen, with Relu and SoftMax serving as activation functions. The DNNs were trained and validated using a batch size of 32 and 200 Epochs. The RNN achieved the highest accuracy rate of 98 % for binary cross-entropy loss. The DT algorithm had an accuracy rating of 97 %, while GB demonstrated the highest Precision, Recall, and f1 scores of 96 %, 98 %, and 97 %, respectively. Our study's promising results suggest the potential for using AI techniques to generate useful clinical information for genomics biomarkers. Future work will explore other omics technologies to predict different diseases. Furthermore, the findings and methodologies presented in this research hold the potential to be seamlessly integrated into practical, real-time scenarios, offering tangible benefits to the field of healthcare at large. This research assesses the performance of various ML and DL classifiers on a single dataset. However, our proposed scheme can be further evaluated on complex datasets. Finally, this research is limited to leveraging ML and DL using a simulation-based approach, the subsequent evaluation in real-time scenarios in dynamic environments can be evaluated.

## Funding statement

The authors are thankful to the Deanship of Scientific Research and the supervision of the Research Center Funding Program at 10.13039/501100005911Najran University for funding this work under the grant code NU/RCP/SERC/12/16.

## Data availability statement

The data associated with our study have been deposited in a publicly available repository: https://github.com/EZubedi/Mutli-omics-integration-for-Leukemia-Prediction.

## CRediT authorship contribution statement

**Erum Zubedi:** Writing – review & editing, Writing – original draft, Methodology, Formal analysis, Conceptualization. **Zhongliang Deng:** Writing – review & editing, Writing – original draft, Resources, Methodology, Formal analysis, Data curation. **Qasim Ali:** Writing – review & editing, Writing – original draft, Methodology, Formal analysis, Data curation. **Adil Khan:** Writing – review & editing, Writing – original draft, Software, Resources, Formal analysis, Data curation. **Asadullah Shaikh:** Writing – review & editing, Supervision, Software, Resources, Methodology, Investigation. **Mana Saleh Reshan:** Writing – review & editing, Visualization, Software, Methodology, Formal analysis, Data curation. **Adel Sulaiman:** Writing – review & editing, Validation, Methodology, Investigation, Formal analysis, Data curation. **Hani Alshahrani:** Writing – review & editing, Resources, Methodology, Investigation, Data curation.

## Declaration of competing interest

The authors declare that they have no known competing financial interests or personal relationships that could have appeared to influence the work reported in this paper.

## References

[1] Araaf M.A., Nugroho K., Setiadi D.R.I.M. (Sep. 2023). Comprehensive analysis and classification of skin diseases based on image texture features using K-nearest neighbors algorithm. J. Comput. Theories Appl..

[2] Picard M., Scott-Boyer M.-P., Bodein A., Périn O., Droit A. (2021). Integration strategies of multi-omics data for machine learning analysis. Comput. Struct. Biotechnol. J..

[3] Santiago-Rodriguez T.M., Hollister E.B. (Oct. 2021). Multi ‘omic data integration: a review of concepts, considerations, and approaches. Semin. Perinatol..

[4] Zheng J. (2020). Integrative analysis of multi-omics identified the prognostic biomarkers in acute myelogenous leukemia. Front. Oncol..

[5] Chu X., Zhang B., Koeken V.A.C.M., Gupta M.K., Li Y. (2021). Multi-omics approaches in immunological research. Front. Immunol..

[6] Dai X., Shen L. (2022). Advances and trends in omics technology development. Front. Med..

[7] Zenbout I., Bouramoul A., Meshoul S., Amrane M. (2023). Efficient bioinspired feature selection and machine learning based framework using omics data and biological knowledge data bases in cancer clinical endpoint prediction. IEEE Access.

[8] Vandereyken K., Sifrim A., Thienpont B., Voet T. (2023). Methods and applications for single-cell and spatial multi-omics. Nat. Rev. Genet. Nature Research.

[9] Haas R., Zelezniak A., Iacovacci J., Kamrad S., Townsend S.J., Ralser M. (2017). Designing and interpreting ‘multi-omic’ experiments that may change our understanding of biology. Curr. Opin. Struct. Biol..

[10] Leng D. (2022). A benchmark study of deep learning-based multi-omics data fusion methods for cancer. Genome Biol..

[11] Nativio R. (Oct. 2020). An integrated multi-omics approach identifies epigenetic alterations associated with Alzheimer's disease. Nat. Genet..

[12] Boehm K.M., Khosravi P., Vanguri R., Gao J., Shah S.P. (2022). Harnessing multimodal data integration to advance precision oncology. Nat. Rev. Cancer.

[13] Zhang W., Lin Z. (2023). iPoLNG—an unsupervised model for the integrative analysis of single-cell multiomics data. Front. Genet..

[14] Sapra L., Sandhu J.K., Goyal N. (2021).

[15] Gulati S., Guleria K., Goyal N. (2022). 2022 2nd International Conference on Advance Computing and Innovative Technologies in Engineering (ICACITE).

[16] Sunarjo M.S., Gan H.-S., Setiadi D.R.I.M. (2023). High-performance convolutional neural network model to identify COVID-19 in medical images. J. Comput. Theories Appl..

[17] Wang T. (2021). MOGONET integrates multi-omics data using graph convolutional networks allowing patient classification and biomarker identification. Nat. Commun..

[18] Verma V. (May 2022). A deep learning-based intelligent garbage detection system using an unmanned aerial vehicle. Symmetry (Basel).

[19] Aggarwal M., Khullar V., Goyal N. (2022). 2022 10th International Conference on Reliability, Infocom Technologies and Optimization (Trends and Future Directions*) (ICRITO)*.

[20] Aggarwal M., Khullar V., Goyal N. (2023). 2023 3rd International Conference on Innovative Practices in Technology and Management (ICIPTM).

[21] Imanulloh S.B., Muslikh A.R., Setiadi D.R.I.M. (2023). Plant diseases classification based leaves image using convolutional neural network. Journal of Computing Theories and Applications.

[22] Choudhary K. (2022). Recent advances and applications of deep learning methods in materials science. npj Comput. Mater..

[23] Bhattacharjee A., Murugan R., Goel T. (2022). A hybrid approach for lung cancer diagnosis using optimized random forest classification and K-means visualization algorithm. Health Technol..

[24] Zaghlool S.B., Attallah O. (2022). Proceedings - 2022 IEEE International Conference on Bioinformatics and Biomedicine, BIBM 2022.

[25] Cao Y. (Jan. 2022). Multi-omics analysis based on genomic instability for prognostic prediction in lower-grade glioma. Front. Genet..

[26] Liu X.-Y., Mei X.-Y. (Apr. 2023). Prediction of drug sensitivity based on multi-omics data using deep learning and similarity network fusion approaches. Front. Bioeng. Biotechnol..

[27] Kourou K., Exarchos K.P., Papaloukas C., Sakaloglou P., Exarchos T., Fotiadis D.I. (2021). Applied machine learning in cancer research: a systematic review for patient diagnosis, classification and prognosis. Comput. Struc. Biotechnol. J..

[28] Yu X. (Feb. 2023). Survey of deep learning techniques for disease prediction based on omics data. Hum. Genet..

[29] Bukhari M., Yasmin S., Sammad S., Abd El-Latif A.A. (2022). A deep learning framework for leukemia cancer detection in microscopic blood samples using squeeze and excitation learning. Math. Probl Eng..

[30] Srikantamurthy M.M., Rallabandi V.P.S., Dudekula D.B., Natarajan S., Park J. (Dec. 2023). Classification of benign and malignant subtypes of breast cancer histopathology imaging using hybrid CNN-LSTM based transfer learning. BMC Med. Imag..

[31] Das P.K., V A D., Meher S., Panda R., Abraham A. (2022). A systematic review on recent advancements in deep and machine learning based detection and classification of acute lymphoblastic leukemia. IEEE Access.

[32] Li J.-F., Ma X.-J., Ying L.-L., Tong Y., Xiang X. (2021). Multi-omics analysis of acute lymphoblastic leukemia identified the methylation and expression differences between BCP-all and T-ALL. Front. Cell Dev. Biol..

[33] Kantarjian H. (2021). Acute myeloid leukemia: current progress and future directions. Blood Cancer J..

[34] James A.R. (Dec. 2019). Long non-coding RNAs defining major subtypes of B cell precursor acute lymphoblastic leukemia. J. Hematol. Oncol..

[35] Bornhäuser M. (2023). Allogeneic hematopoietic cell transplantation vs standard consolidation chemotherapy in patients with intermediate-risk acute myeloid leukemia. JAMA Oncol..

[36] Gibson B.E.S., Sauer M.G., Amrolia P. (2019). The EBMT Handbook.

[37] Schroeder M.P. (2019). Integrated analysis of relapsed B-cell precursor Acute Lymphoblastic Leukemia identifies subtype-specific cytokine and metabolic signatures. Sci. Rep..

[38] Leo I.R. (2022). Integrative multi-omics and drug response profiling of childhood acute lymphoblastic leukemia cell lines. Nat. Commun..

[39] Shaikh F.J., Rao D.S. (2021). Materials Today: Proceedings.

[40] Saleh H., Abd-el ghany S.F., Alyami H., Alosaimi W. (2022). Predicting breast cancer based on optimized deep learning approach. Comput. Intell. Neurosci..

[41] Chen S. (2022). Models of artificial intelligence-assisted diagnosis of lung cancer pathology based on deep learning algorithms. J Healthc Eng.

[42] Sarker I.H. (2021). Deep learning: a comprehensive overview on techniques, taxonomy, applications and research directions. SN Comput. Sci..

[43] Gao F., Huang K., Xing Y. (Oct. 2022). Artificial intelligence in omics. Dev. Reprod. Biol..

[44] Zhang X., Zhang J., Sun K., Yang X., Dai C., Guo Y. (Aug. 2019).

[45] H. Chai, X. Zhou, Z. Zhang, J. Rao, H. Zhao, and Y. Yang, “Integrating Multi-Omics Data through Deep Learning for Accurate Cancer Prognosis Prediction”, doi: 10.1101/807214. 10.1016/j.compbiomed.2021.104481 33989895

[46] Sharifi-Noghabi H., Zolotareva O., Collins C.C., Ester M. (2019). Bioinformatics.

[47] Huang Z. (2019). Salmon: survival analysis learning with multi-omics neural networks on breast cancer. Front. Genet..

[48] Yang H., Chen R., Li D., Wang Z. (2021). Subtype-GAN: a deep learning approach for integrative cancer subtyping of multi-omics data. Bioinformatics.

[49] Viaud G., Mayilvahanan P., Cournede P.H. (2022). Representation learning for the clustering of multi-omics data. IEEE ACM Trans. Comput. Biol. Bioinf.

[50] Ponzi E., Thoresen M., Haugdahl Nøst T., Møllersen K. (2021). Integrative, multi-omics, analysis of blood samples improves model predictions: applications to cancer. BMC Bioinf..

[51] Xu X., Gu H., Wang Y., Wang J., Qin P. (2019). Autoencoder based feature selection method for classification of anticancer drug response. Front. Genet..

[52] Zamanian C. (2022). Systems neuroimmunology: a review of multiomics methodologies to characterize neuroimmunological interactions in spinal and cranial diseases. Neurosurg. Focus.

[53] Zhang L., Yang Y., Wang Z., Li D., Liu J., Yang H. (2022). Proceedings - 2022 IEEE International Conference on Bioinformatics and Biomedicine, BIBM 2022.

[54] Siebert J.C., Saint-Cyr M., Borengasser S.J., Wagner B.D., Lozupone C.A., Görg C. (Dec. 2021). CANTARE: finding and visualizing network-based multi-omic predictive models. BMC Bioinf..

[55] Zhou M. (Jun. 2021). Development and evaluation of a leukemia diagnosis system using deep learning in real clinical scenarios. Front Pediatr.

[56] Cheng L.H., Hsu T.C., Lin C. (2021). Integrating ensemble systems biology feature selection and bimodal deep neural network for breast cancer prognosis prediction. Sci. Rep..

[57] Wang C., Lue W., Kaalia R., Kumar P., Rajapakse J.C. (2022). Network-based integration of multi-omics data for clinical outcome prediction in neuroblastoma. Sci. Rep..

[58] El-Manzalawy Y. (2018). 2018 IEEE Conference on Computational Intelligence in Bioinformatics and Computational Biology (CIBCB).

[59] Ma T., Zhang A. (2018). 2018 IEEE International Conference on Bioinformatics and Biomedicine (BIBM).

[60] ICGC Data Portal, a public repository, https://dcc.icgc.org/, https://dcc.icgc.org/releases/current/Projects/ALL-US.

[61] Flores J.E., Claborne D.M., Weller Z.D., Webb-Robertson B.-J.M., Waters K.M., Bramer L.M. (2023). Missing data in multi-omics integration: recent advances through artificial intelligence. Front Artif. Intell..

[62] Wongoutong C. (2022). Imputation methods for missing response values in the three parts of a central composite design with two factors. J. Stat. Comput. Simulat..

[63] Yi X., Xu Y., Hu Q., Krishnamoorthy S., Li W., Tang Z. (Jun. 2022). ASN-SMOTE: a synthetic minority oversampling method with adaptive qualified synthesizer selection. Complex and Intelligent Systems.

[64] Putri G.H., Anders S., Pyl P.T., Pimanda J.E., Zanini F. (2022). Analysing high-throughput sequencing data in Python with HTSeq 2.0. Bioinformatics.

[65] Becker M. (2023). Large-scale correlation network construction for unraveling the coordination of complex biological systems. Nat. Comput. Sci..

[66] Senthilnathan S. (Jul. 2019). Usefulness of correlation analysis. SSRN Electron. J..

[67] Allesøe R.L. (Mar. 2023). Discovery of drug–omics associations in type 2 diabetes with generative deep-learning models. Nat. Biotechnol..

[68] Alhenawi E., Al-Sayyed R., Hudaib A., Mirjalili S. (2022). Feature selection methods on gene expression microarray data for cancer classification: a systematic review. Comput. Biol. Med..

[69] Huang W., Tan K., Hu J., Zhang Z., Dong S. (2022). A review of fusion methods for omics and imaging data. IEEE ACM Trans. Comput. Biol. Bioinf.

[70] Hosseinpour M., Ghaemi S., Khanmohammadi S., Daneshvar S. (2022). A hybrid high‐order type‐2 FCM improved random forest classification method for breast cancer risk assessment. Appl. Math. Comput..

[71] Pfeifer B., Baniecki H., Saranti A., Biecek P., Holzinger A. (2022). Multi-omics disease module detection with an explainable Greedy Decision Forest. Sci. Rep..

[72] Subasree S., Sakthivel N.K., Tripathi K., Agarwal D., Tyagi A.K. (2022). Combining the advantages of radiomic features based feature extraction and hyper parameters tuned RERNN using LOA for breast cancer classification. Biomed. Signal Process Control.

[73] Admon M.R., Senu N., Ahmadian A., Abdul Majid Z., Salahshour S. (2023). A new efficient algorithm based on feedforward neural network for solving differential equations of fractional order. Commun. Nonlinear Sci. Numer. Simul..

[74] Miron M., Moldovanu S., Culea-Florescu A.L. (2022). 2022 26th International Conference on System Theory, Control and Computing (ICSTCC).

[75] Feldner-Busztin D. (2023). Dealing with dimensionality: the application of machine learning to multi-omics data. Bioinformatics.

[76] Alsenan S., Al-Turaiki I., Hafez A. (2020). A Recurrent Neural Network model to predict blood–brain barrier permeability. Comput. Biol. Chem..

[77] Agarap A.F. (2018). http://arxiv.org/abs/1803.08375.

[78] Rong Z., Lingyun D., Jinxing L., Ying G. (2021). Diagnostic classification of lung cancer using deep transfer learning technology and multi‐omics data. Chin. J. Electron..

[79] Tian Y., Su D., Lauria S., Liu X. (2022). Recent advances on loss functions in deep learning for computer vision. Neurocomputing.

[80] Wang Q., Ma Y., Zhao K., Tian Y. (2022). A comprehensive survey of loss functions in machine learning. Annals of Data Science.

[81] Wang W., Lu Y. (2018). IOP Conference Series: Materials Science and Engineering.

[82] Hurtik P., Tomasiello S., Hula J., Hynar D. (Jul. 2022). Binary cross-entropy with dynamical clipping. Neural Comput. Appl..

[83] Arjmand B. (2022). Machine learning: a new prospect in multi-omics data analysis of cancer. Front. Genet..

[84] ElKarami B., Alkhateeb A., Qattous H., Alshomali L., Shahrrava B. (2022). Multi-omics data integration model based on UMAP embedding and convolutional neural network. Cancer Inf..

[85] Rong Z. (2022). MCluster-VAEs: an end-to-end variational deep learning-based clustering method for subtype discovery using multi-omics data. Comput. Biol. Med..

[86] Alkhateeb A. (2022). 2022 *21st* IEEE International Conference on Machine Learning and Applications (ICMLA).

[87] Zhou X.-J., Zhong X.-H., Duan L.-X. (2023). Integration of artificial intelligence and multi-omics in kidney diseases. Fundamental Research.

[88] Azmi N.S. (2022). Comparative analysis of deep learning algorithm for cancer classification using multi-omics feature selection. Progress In Microbes & Molecular Biology.

[89] Bagante F. (2021). Artificial neural networks for multi-omics classifications of hepato-pancreato-biliary cancers: towards the clinical application of genetic data. Eur. J. Cancer.

[90] Hu Y., Zhao L., Li Z., Dong X., Xu T., Zhao Y. (2022). Classifying the multi-omics data of gastric cancer using a deep feature selection method. Expert Syst. Appl..

[91] Asada K. (2020). Uncovering prognosis-related genes and pathways by multi-omics analysis in lung cancer. Biomolecules.

[92] Zhang Z., Wei Z., Zhao L., Gu C., Meng Y. (Dec. 2023). Assessing the clinical utility of multi-omics data for predicting serous ovarian cancer prognosis. J. Obstet. Gynaecol. (Lahore).

[93] Ma B., Meng F., Yan G., Yan H., Chai B., Song F. (2020). Diagnostic classification of cancers using extreme gradient boosting algorithm and multi-omics data. Comput. Biol. Med..

